# A nomogram to predict “pure” vs. “mixed” uric acid urinary stones

**DOI:** 10.1007/s00345-024-05340-3

**Published:** 2024-10-31

**Authors:** Liran Zieber, Gherman Creiderman, Muhammad Krenawi, Daniel Rothenstein, Dmitry Enikeev, Yaron Ehrlich, David Lifshitz

**Affiliations:** 1 Institute of Urology, Rabin Medical Center, Petah Tikva, Israel; 2 Faculty of Medical and Health Sciences, Tel-Aviv University, Tel Aviv, Israel; 3 Urology Department, Medical University of Vienna, Vienna, Austria

**Keywords:** Computed tomography, Nephrolithiasis, Nomogram, Stone composition, Uric acid

## Abstract

**Purpose:**

Uric acid stones (UAS) can be treated non-invasively by oral chemolysis. However, it is crucial to identify individuals who are most likely to benefit from this approach, specifically, patients with pure UAS. The aim of this study was to develop a nomogram that can differentiate between pure and mixed UAS.

**Methods:**

A retrospective analysis of demographic, clinical and stone composition data of patients with a predominant UAS composition (≥ 50%) treated between 2014 and 2022.

**Results:**

A total of 135 patients were included in the analysis, 37.8% had mixed UAS (50–90% UA) and 62.2% had pure UAS (≥ 95% UA). The mean stone density and the percentage of radiopaque stones in the pure UAS group were significantly lower than those in the mixed UAS group (450 Hounsfield Units [HU] vs. 600 HU, and 24% vs. 58%, respectively). A stepwise multivariate logistic regression revealed that lower stone density, bigger size, decreased stone opacity and older age are predictive variables for pure UAS. Accordingly, a nomogram was generated with a receiver operating characteristic (ROC) curve that showed an area under the curve (AUC) of 0.78. A patient with a total score of 156 has a probability of > 95% for pure UAS.

**Conclusion:**

Imaging and demographic data can be used to identify patients with pure UAS. The nomogram may be useful for counseling patients regarding oral chemolysis. Future validation of the nomogram with a different data set is required to assess its efficacy.

## Introduction

The prevalence of uric acid stones (UAS) is 8–10% globally with an increasing prevalence worldwide [1]. The percentage of UAS among all urinary tract stones has increased in recent decades. For example, a retrospective study conducted in the USA has reported an increase from 7 to 14% between 1980 and 2015 [2], whereas in Norway an increase from 2.0 to 9.1% was reported over a 40-year period [3]. Risk factors for the formation of UAS include traits of the metabolic syndrome, particularly insulin resistance, obesity and hypertension [4–10]. UAS formers were consistently found to be older than other stone formers with their proportion rising to 21% among patients over 60 years of age [2, 10, 11]. Additionally, UAS formers have a higher recurrence rate and a shorter recurrence interval, as compared to patients with calcium stones [12].

UAS can be treated non-invasively by oral chemolysis, which involves the administration of oral medications that chemically dissolve the stones within the urinary tract by altering urine pH, typically through the use of alkalizing agents like potassium citrate or sodium bicarbonate. However, patients are required to adhere to an extensive medication regime for a period of several months and treatment success, which varies widely, ranging from 34 to 83% [13–18], is not guaranteed [14]. Predicting the success rate of oral chemolysis is challenging because a previous stone analysis is often unavailable and stone composition is estimated by clinical and imaging data. Although various methods and predictive models to identify UAS exist [19–26], they were mostly based on studies that differentiated between calcium oxalate stones and UAS. However, only a minority of the studies provided information about the actual composition of the UAS, usually defined as stones with a predominant (> 50%) UA component. Some utilized demographic and clinical parameters, including urine sample results [19, 20, 27]. Others combined various CT techniques, showing that UAS can be identified based on radiodensity values ranging from 200 to 632 Hounsfield units (HU) [21–26]. However, such broad definitions are bound to include both pure UAS, which are more likely to dissolve via oral chemolysis, and mixed, heterogeneous stones which are less likely to completely dissolve by this treatment.

To improve the selection process of suitable candidates for oral chemolysis, we aimed to develop a novel nomogram that may assist in differentiating between pure UAS consisting of ≥ 95% UA and mixed UAS comprising 50-90% UA.

## Materials and methods

### Study design and setting

We have retrospectively reviewed a database containing information on all urinary tract stones analyzed at our institute between 2014 and 2022. The stones were retrieved during surgical procedures or were passed spontaneously. The study was approved by the institutional ethics committee (approval number RMC-0747-21, 22 November 2021).

### Patients

Patients over 18 years of age with predominant UAS (≥ 50%) composition and preoperative non-contrast CT scans were included in the study. Patients with a stone diameter of less than 5 mm were excluded.

Patients’ demographic and clinical data included age, gender, BMI, comorbidities such as hypertension, diabetes, serum calcium, serum UA, glycosylated hemoglobin (HbA1c), urinary pH and the concentration of UA in urine collected over 24 h.

### Stone composition analysis

The stones were analyzed using Fourier Transform Infrared Spectroscopy (ALPHA FTIR spectrometer, Bruker, Karlsruhe, Germany) and the results were reported in 5% increments.

CT attenuation characteristics and stone size were measured in the coronal and axial views. The region of interest (ROI) was drawn in the central area of the stone. Stone opacity was classified as either radiopaque or radiolucent, depending on whether it was visible or not, according to abdominal X-rays or a CT scout (topogram). Patients were classified based on stone composition into two groups: mixed UAS (50–90% UA) and pure UAS (95–100% UA).

### Statistical analysis

Statistical analysis was performed using SAS^®^ version 9.4 (SAS Institute, Cary, NC, USA). Continuous variables were summarized by mean and standard deviation (SD) and compared using the Wilcoxon-Mann Whitney test and Student’s T test. Categorical variables were summarized by number and frequency (%) and compared using Fisher’s exact test and chi-squared test.

A stepwise logistic regression was performed to find the best predictors for pure UAS and create a model. A receiver operating characteristics (ROC) curve was constructed to evaluate the goodness of fit of the model. A nomogram that graphically represents the numerical relationships between the predictors for pure UAS was constructed using the logistic regression model.

## Results

A total of 135 patients were included in the analysis, 51 (37.8%) had mixed UAS and 84 (62.2%) had pure UAS. Table 1 compares the clinical, demographic and stone characteristics of the two groups. Except for diabetes, whose rate was significantly higher in the pure UAS group compared to the mixed UAS group (63% vs. 41%, *p* = 0.013), all other metabolic and demographic parameters were similar in the two groups.

**Table 1:** Demographic, clinical and stone characteristics of the study population by stone composition

	Mixed UAS (50–90%) *N* = 51	Pure UAS (95–100%) *N* = 84	*P*-value
Age (years)	60 (± 11)	63 (± 13)	0.14
Sex			
Men	38 (75%)	55 (65%)	0.27
Women	13 (25%)	29 (35%)	
Comorbidities			
Diabetes	21 (41%)	53 (63%)	0.013
Hypertension	30 (59%)	55 (65%)	0.44
Hyperlipidemia	21 (41%)	40 (48%)	0.47
Glycosylated hemoglobin (%)	6.89 (± 1.32)	7.17 (± 1.46)	0.37
Body mass index (kg/m^2^)	30 (± 5)	31 (± 6)	0.13
Urinary pH	5.39 (± 0.39)	5.37 (± 0.43)	0.78
Urine uric acid (mg/24 h)	635 (± 286.53)	585 (± 268.84)	0.49
Serum uric acid (mg/dl)	6.56 (± 1.57)	6.97 (± 1.71)	0.15
Serum Calcium (mg/dl)	9.31 (± 0.55)	9.28 (± 0.47)	0.77
Creatinine base (mg/dl)	1.11 (± 0.43)	1.1 (± 0.62)	0.87
Mean Stone Density (HU) (IQR)	600 (400–897)	450 (393–500)	< 0.0001
Radiopaque Stones	30 (58%)	20 (24%)	0.0002
Stone Size (mm) (IQR)	15 (10–17)	15 (8–20)	0.87

Continuous variables are presented as mean (± standard deviation) and categorical variables are presented as number and frequency (%). IQR = interquartile range

The secondary components in the mixed UAS group were calcium oxalate, ammonium urate and sodium urate in 88%, 8% and 4% of the stones, respectively. The mean stone density and the percentage of radiopaque stones among patients with pure UAS were significantly lower than in the mixed UAS group, with values of 450 HU vs. 600 HU and 24% vs. 58%, respectively (*p* ≤ 0.0002) (Table 1). The mean stone size was 15 mm in both groups and the size of radiolucent kidney stones was similar in both groups. However, radio opacity was associated with significantly larger stones in the pure UAS group in comparison to the mixed UAS group (33 mm vs. 20 mm, *p* = 0.02).

**Fig. 1:**
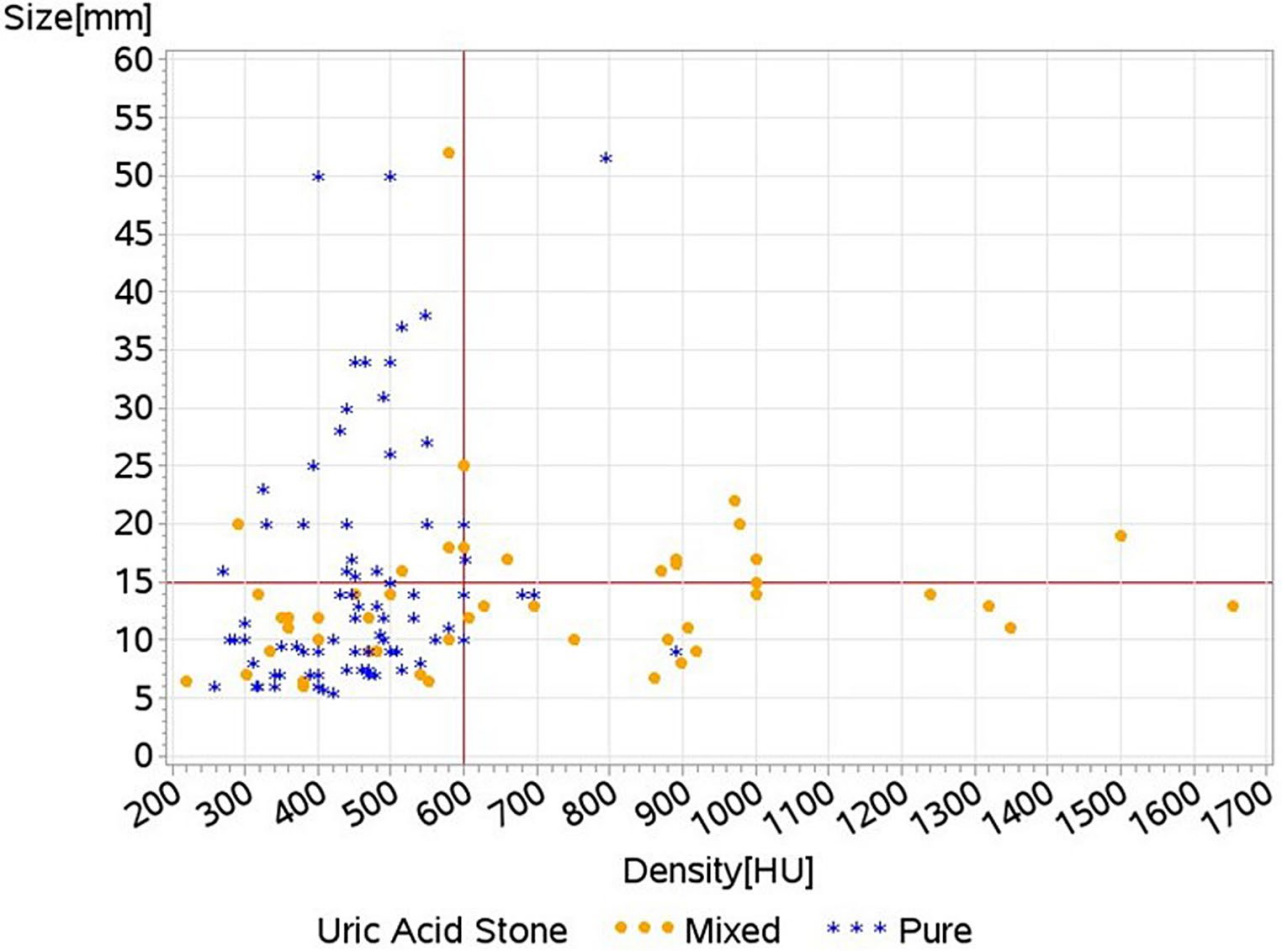
Stone size vs. stone density by uric acid stone type. A scatter plot of UAS size vs. density. The best predictive cutoff is achieved with stone density lower than 600 HU and stone size larger than 15 mm. Stones within these ranges have a 77.4% probability for having a pure UA composition (≥ 95%) (*p* = 0.0023)

Significant predictors for pure UAS in a univariate analysis were found to be stone density (*p* < 0.0001) and stone opacity (*p* = 0.0002). Analysis of the predictive accuracy of stone density showed it was affected by stone size. Stones with a density under 600 HU and a size greater than 15 mm had 77.4% likelihood of being pure UA (*p* = 0.0023). This probability decreased to 71% for stones smaller than 15 mm (*p* = 0.0005), as illustrated in Fig. 1.

**Fig. 2:**
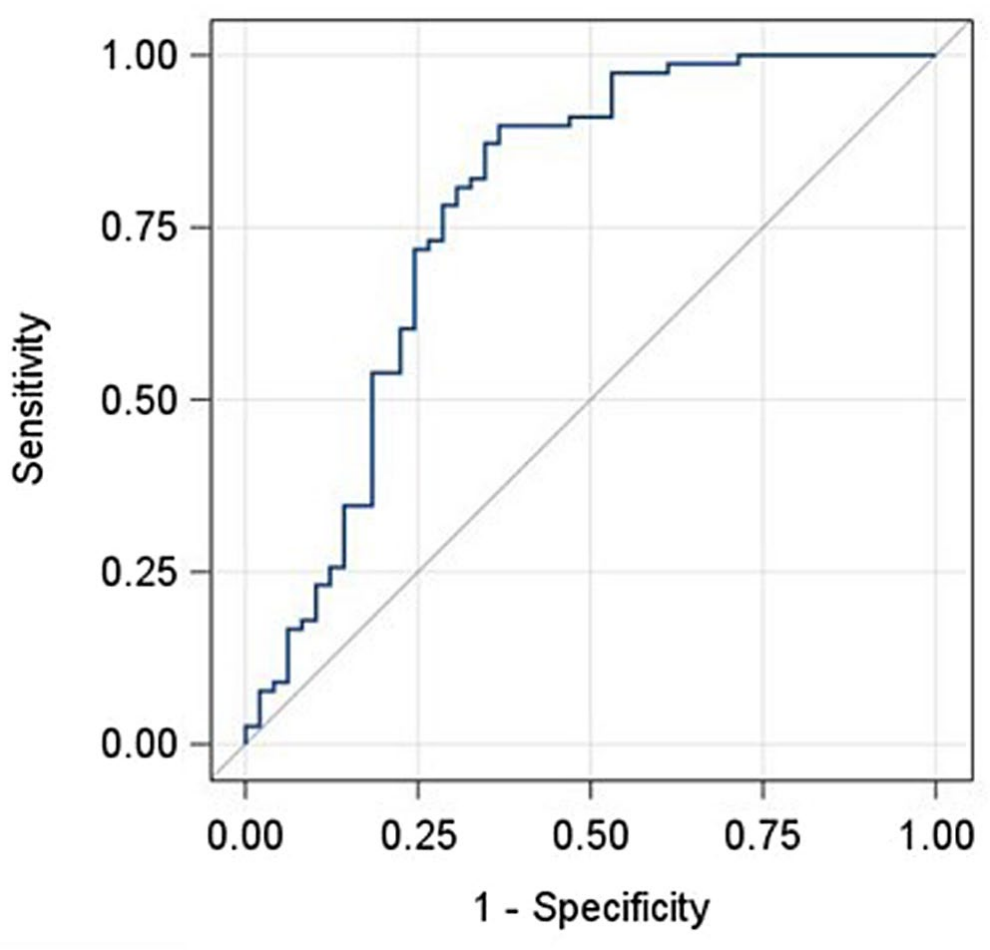
A receiver operating characteristics (ROC) curve showing the model’s goodness of fit. The area under the curve is 0.78

A stepwise multivariate logistic regression analysis (Table 2) revealed that in addition to lower stone density and radiolucency, larger stones and older age were predictive variables for pure UAS. The model’s goodness of fit incorporating these variables is shown by the ROC curve in Fig. 2, with an area under the curve (AUC) of 0.78. Other variables, including the presence of diabetes, HbA1c and serum UA levels were not predictors of stone composition.

**Table 2:** Multivariate binary logistic regression to predict uric acid stone composition

Parameter	*P*-value	Odds ratio (95% CI)	Regression coefficient
Density (HU)	0.0017	0.995 (0.993–0.998)	-0.005
Size (mm)	0.0213	1.065 (1.009–1.123)	0.063
Opacity (radiolucent)	0.0723	2.671 (0.915–7.800)	0.983
Age (years)	0.0468	1.034 (1.000–1.068)	0.033

CI = confidence interval

**Fig. 3:**
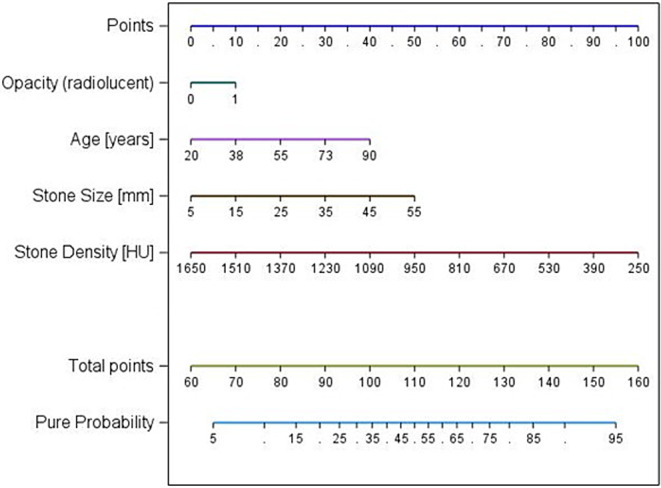
Nomogram for predicting pure uric acid stones. The scores on the horizontal lines reflect the association of a given factor with the probability of a stone with a pure UA composition. The sum of the four predictors (stone opacity, size, density and age) constitutes the total score that translates to the probability of a pure UAS for a given patient

Next, a nomogram was constructed using the stepwise logistic regression model (Fig. 3). The scores on the horizontal lines reflect the association of a given factor with the probability of a stone with a pure UA composition. The sum of the four predictors (stone opacity, size, density and age) constitutes the total score that translates to the probability of a pure UAS for a given patient. With a total score of 156 the probability for a pure UAS is > 95%.

## Discussion

In the current study we developed, for the first time, a nomogram designed to help differentiate between pure and mixed UAS based on both demographic and imaging variables. The nomogram includes stone density, stone opacity, stone size and patient age. The model’s ROC curve has an AUC of 0.78. Torricelli et al. [19, 27] generated a nomogram designed to distinguish between uric acid and calcium oxalate stones based on 24-hour urine collection parameters, BMI and patient age. Their ROC curve showed a relatively good AUC of 0.8 with the best cutoff score providing high sensitivity but poor specificity (87.5% and 68%, respectively). The various proportions of UAS in the study may explain the low specificity observed. Clearly, adding imaging data to clinical and demographic data would improve any prediction model.

Traditionally, stone radiolucency was one of the characteristics suggestive of UAS. In recent years, clinicians tend to rely more on stone density as determined by CT. Multiple studies have shown the predictive value of CT and more recently, dual energy CT, in predicting UAS composition [21–26]. Interestingly, we have found that stone opacity adds accuracy to a model that already includes stone density. Likewise, Wiessmeyer et al. [20] has recently presented a nomogram that incorporates clinical as well as imaging data to predict UAS vs. non-UAS composition of ureteral stones. Their data included 17 patients with pure UAS and 28 patients with mixed UAS. In addition to age, BMI, serum UA and urine pH, stone density as well as stone opacity were independent predictors for UAS composition. The rate of radiolucency was higher for UAS in comparison to non-UAS (88.6% vs. 32.7%, *p* < 0.001), and stone density was lower (435 HU vs. 750 HU, *p* < 0.001). As expected, the differences in opacity and stone density in our study, which examined pure and mixed UAS and excluded calcium oxalate stones, were smaller (76% vs. 42%, *p* = 0.0002 and 450 vs. 600 HU, *p* < 0.0001, respectively). Although Wiessmeyer et al. [20] combined pure and mixed (> 50%) UAS in the same group, the mean stone density of pure UAS in their study, 435 HU, was similar to that found in the current study (450 HU). This may be explained by their inclusion of a relatively large cluster of smaller stones (33% were < 5 mm). It has been shown that the accuracy of stone density measurements is diminished in smaller stones, generating lower HU values than would otherwise be expected [22, 28, 29]. Indeed, our analysis showed that prediction accuracy was better for larger stones as expressed in the nomogram (Fig. 3). Clearly, it is easier to define opacity in larger stones. Moreover, density measurements are less likely to include non-stone areas. Thus, stone size was found to be one of the predictors in the nomogram.

A few studies have evaluated the differences in clinical and demographic characteristics between pure and mixed UAS formers. Friedlander et al. [10] compared the metabolic profile of pure UAS, pure calcium oxalate stone and mixed stone (defined as at least 10% of each component) formers. Similar to our results, the rate of diabetes among patients with pure UAS was almost twice that of patients with mixed stones. Likewise, Fernandez et al. [30] found that pure UAS were more prevalent among patients with diabetes compared to patients without diabetes (63% vs. 46%). Reichard et al. [31] concluded that older and heavier patients with higher serum UA levels are more likely to have pure UAS. We found no differences in mean daily urinary UA excretion between pure UAS and mixed UAS formers, and a non-statistically significantly higher serum UA in the pure UAS group. Therefore, it seems that only a combination of clinical, demographic and imaging data might improve the prediction of pure UAS.

The limitations of the study include its retrospective design. In addition, like other clinical studies evaluating stone composition, stone analysis was based on collected stones which may not represent the actual proportion of UAS among non-fragmented stones. Although our cohort did not include radiolucent stones of different compositions such as cystine or struvite stones, the rarity of cystine stones and the associated distinct clinical features of infection stones, may not decrease the model’s function.

## Conclusion

Predicting pure UAS, which are more likely to respond to chemolysis, is feasible using a nomogram based on readily available data. The nomogram may be useful for counseling patients regarding oral chemolysis. Future validation of the nomogram with a different data set is required to assess its efficacy.

## Data Availability

The data that support the findings of this study are available from the corresponding author upon reasonable request.
